# Exploring impaired self-awareness of motor symptoms in Parkinson’s disease: Resting-state fMRI correlates and the connection to mindfulness

**DOI:** 10.1371/journal.pone.0279722

**Published:** 2023-02-24

**Authors:** Timo Marcel Buchwitz, Marina Christine Ruppert-Junck, Andrea Greuel, Franziska Maier, Franziska Thieken, Viktoria Jakobs, Carsten Eggers

**Affiliations:** 1 Department of Neurology, University Hospital Marburg, Marburg, Germany; 2 Center for Mind, Brain, and Behavior (CMBB), Universities Marburg and Gießen, Marburg, Germany; 3 Department of Psychiatry, University Hospital Cologne, Medical Faculty, Cologne, Germany; 4 Department of Neurology, Knappschaftskrankenhaus Bottrop GmbH, Bottrop, Germany; Carl von Ossietzky Universitat Oldenburg, GERMANY

## Abstract

**Objective:**

To further explore the phenomenon of impaired self-awareness of motor symptoms in patients with Parkinson’s Disease by using an evaluated measurement approach applied in previous studies, while also examining its connection with dispositional mindfulness and possible correlates of functional connectivity.

**Background:**

Recently, the phenomenon of impaired self-awareness has been studied more intensively by applying different measurement and imaging methods. Existing literature also points towards a possible connection with mindfulness, which has not been examined in a cross-sectional study. There is no data available concerning correlates of functional connectivity.

**Methods:**

Non-demented patients with idiopathic Parkinson’s Disease without severe depression were tested for impaired self-awareness for motor symptoms following a psychometrically evaluated approach. Mindfulness was measured by applying the German version of the Five Facet Mindfulness Questionnaire. A subset of eligible patients underwent functional MRI scanning. Spearman correlation analyses were performed to examine clinical data. Whole-brain voxelwise regressions between seed-based connectivity and behavioral measures were calculated to identify functional connectivity correlates of impaired self-awareness scores.

**Results:**

A total of 41 patients with Parkinson’s Disease were included. 15 patients successfully underwent resting-state fMRI scanning. Up to 88% of patients showed signs of impaired self-awareness. Awareness for hypokinetic movements correlated with total mindfulness values and three facets, while awareness for dyskinetic movements did not. Three significant clusters between scores of impaired self-awareness in general and for dyskinetic movements were identified linking behavioral measures with the functional connectivity of the inferior frontal gyrus, the right insular cortex, the supplementary motor area, and the precentral gyrus among others. Impaired self-awareness for hypokinetic movements did not have any neural correlate.

**Conclusions:**

Clinical data is comparable with results from previous studies applying the same structured approach to measure impaired self-awareness in Parkinson’s Disease. Functional connectivity analyses were conducted for the first time to evaluate neural correlates thereof. This data does not support a connection between impaired self-awareness of motor symptoms and dispositional mindfulness.

## Introduction

Self-awareness is defined as a complex multidimensional phenomenon of “becoming the object of one’s own attention” [1]. Impaired self-awareness (ISA) has been repeatedly reported, especially in regard to perceiving one’s own neurological and neuropsychological symptoms [2]. In Parkinson’s Disease (PD), research has mainly focused on the phenomenon of impaired self-awareness of motor symptoms (ISAm) [3].

Previous studies reported a varying incidence of ISAm ranging from approximately 23% [4] to a total of 66% [5] in samples of PD patients. However, these frequencies comprise data including different kinds of ISAm, which was also acquired by using various measurement methods. Due to the assumption of different underlying pathological mechanisms, a reasonable discrimination between ISAm for levodopa-induced dyskinesias (ISAm-LID) and ISAm for hypokinetic movements (ISAm-Hypo), e.g. bradykinesia, has become more common in research [6]. For the purpose of completeness, it is worth mentioning, that to the best of our knowledge, only one study has investigated the functional correlates of general ISAm in PD patients. Using 18F-fluorodeoxyglucose positron emission tomography (FDG-PET), Maier et al. (2016) found a correlation between higher ISAm-total and higher metabolism in the bilateral medial frontal gyrus, the left inferior frontal gyrus (IFG), the right superior frontal gyrus and the right precentral gyrus in PD patients with adequate dopaminergic medication [7]. These frontal regions have previously been linked to ISA of cognitive deficits in Alzheimer’s Disease [8].

In previous studies, ISAm-Hypo was present in up to 54% of PD patients, when measured in the ON-state with adequate dopaminergic medication, while no significant clinical or neurobiological correlates were identified [5, 7]. However, due to strong correlation between ISAm-Hypo in the medication ON- and OFF-state, similar underlying mechanisms were suggested. Relevant clinical correlates of ISAm-Hypo might be older age and shorter disease duration. Similar to anosognosia in stroke patients, ISAm-Hypo is assumed to be caused by right hemispheric dysfunction [2, 9]. The right IFG and the right insular cortex (IC) might be important brain regions of interest, as they are part of a network, which was associated with motor inhibition and action monitoring [10].

In contrast, ISAm-LID was found in up to 91% of patients with dyskinesias [5, 11]. A higher disease duration, higher levodopa equivalent daily dose (LEDD), and predominantly left-sided symptoms were identified as significant clinical correlates [4, 7, 12]. Furthermore, executive functioning and the general level of cognitive performance were associated with ISAm-LID, although the latter was not supported by all studies [e.g. 13]. It is hypothesized, that PD patients with ISAm-LID struggle with monitoring their own performance and the appropriate use of feedback. Neural mechanisms monitoring planned versus actual movements are malfunctioning, which results in further difficulties to discriminate between accurate and inaccurate execution of planned movements [6]. On a neurobiological level, many regions were linked with unawareness of dyskinesias in previous studies [7, 12–15]. Particularly, increased metabolism in the bilateral mid-cingulate cortex (MCC) and the left paracentral lobule was correlated with ISAm-LID in PD patients in a previous FDG-PET study [7]. In a task-based functional magnetic resonance imaging (fMRI) study, Palermo et al. (2018) highlighted the importance of the anterior cingulate cortex (ACC) as a potential main hub for the interpretation of dyskinetic movements with support of the dorsolateral prefrontal cortex (DLPFC) and the anterior IC. The authors conclude, that the interaction of these regions may be part of a larger system involved in building an “integrated awareness” of physical, affective and cognitive states [13].

### Dispositional mindfulness

Mindfulness can be classically described as “paying attention in a particular way: on purpose, in the present moment, and nonjudgmentally” [16]. Mindfulness in general is associated with many health-related variables, e.g. quality of life and optimism, and negatively associated with psychiatric symptoms like depression or anxiety [17]. In research, mainly the evaluation of behavioral and neurobiological changes caused by regular mindfulness training or meditation (state mindfulness) was emphasized. In contrast, trait or dispositional mindfulness describes an individual’s natural tendency to adopt a mindful perspective across experiences and contexts [e.g. 18, 19]. While state and trait/ dispositional mindfulness might be quite similar in long-term meditators, they have to be differentiated for people naïve to meditation, meaning they never had a regular and extensive meditation practice [20–22].

Several neuroimaging studies have examined dispositional mindfulness and its neural correlates in healthy subjects. For example, in a task-based fMRI study Dickenson et al. (2013) observed, that people with greater dispositional mindfulness scores showed greater activity in the right temporo-parietal junction, superior parietal lobule and DLPFC during a focused breathing task [23]. Lu and colleagues (2014) used voxel-based morphometry in a sample of 247 subjects and linked greater mindfulness with greater gray matter volume in the right hippocampus, amygdala, and bilateral ACC, and with less gray matter volume in the bilateral posterior cingulate cortex and left orbitofrontal cortex. In accordance with the literature, they concluded, that these brain regions were involved in executive functioning, emotion regulation, and self-referential processing [24]. Recently, Parkinson et al. (2019) examined dispositional mindfulness (including its facets) and functional connectivity in cognitive in addition to attentional resting-state networks in healthy subjects (undergraduate students). They highlighted functional connectivity in the ACC and IC, as well as mindfulness-dependent variability of connectivity in the MCC, cerebellum, and sensorimotor regions [18]. They conclude that dispositional mindfulness is therefore related to functional connectivity of regions associated with cognition, emotion, and sensation.

Previous articles pointed out, that mindfulness (meditation) has an influence on self-awareness in general as well as body-related processes like body awareness and interoception [e.g. 25, 26]. Although never explicitly studied before, there appears to be an overlap between mindfulness and ISAm on a conceptual, behavioral, and neurobiological level. Therefore we hypothesize, that mindfulness (or its facets) and ISAm in PD are connected.

### Aims of this study

The aim of this study was to further explore the phenomenon of ISAm in PD, including analyses of its clinical and resting-state fMRI correlates. Also, a possible connection to dispositional mindfulness is probed. To the best of our knowledge, no previous study has investigated resting-state fMRI correlates of ISAm, and the connection between ISAm and dispositional mindfulness in PD. In comparison to previous work by Maier et al. (2016), we examined ISAm and its clinical correlates in a separate group of PD patients [7]. Studies of ISAm often use different approaches to measure this phenomenon. Therefore, we wanted to see, if the results reported by Maier et al. (2016) are replicable, when using the same assessment tool. Moreover, a subsample of our cohort underwent resting-state fMRI to further explore functional connectivity correlates of ISAm. Lastly, we intended to investigate the connection between ISAm and mindfulness in a cross-sectional study using behavioral data from a relatively large, well-characterized cohort.

## Materials and methods

The study has been approved by the local ethics committee of the medical faculty of the Philipps-University of Marburg (study number: 119/18) and has been pre-registered at the German Clinical Trials Register (DRKS00015807). The data presented in this study was obtained as part of a mixed-method, randomized clinical trial comparing an intervention group and a waitlist-control group in a pre-post design to evaluate the effectiveness of a newly developed mindfulness training in PD. In the current study, the clinical, and neurobiological baseline data were analyzed. For a more detailed description see Buchwitz et al. (2020) [27].

### Sample

41 patients with idiopathic PD, diagnosed according to the Movement Disorder Society PD criteria [28], were recruited from regional patient support groups and the Department of Neurology, University Hospital Marburg, Germany. Patients with severe dementia [score <15 in the Parkinson Neuropsychometric Dementia Assessment [29]], severe depression [score >28 in the Beck Depression Inventory-2 (BDI-II) [30, 31]] or additional diagnosis of severe neurological or psychiatric disorders were excluded.

Additional exclusion criteria were left-handedness or ambidexterity, and deep brain stimulation. All patients had no prior experience in meditation, and are therefore described as naïve to meditation. Patients, who fulfilled criteria for an MRI scan, were asked for their willingness to also take part in a structural and functional MRI scan. Clinical and imaging data of patients, who successfully participated in an MRI scan, were used to form a subsample for further analyses. All patients gave written informed consent in accordance with the Declaration of Helsinki.

### Behavioral and clinical measures

All measurements were performed in the medication ON-state. Dose of medication was unchanged for at least two weeks prior to data collection. ISAm, disease severity according to Unified Parkinson’s Disease Rating Scale part III [UPDRS [32]] as well as cognitive functioning were evaluated during in-person meetings. To evaluate ISAm, the structured approach developed by Maier et al. (2015) was used [5]. The approach has been previously explained in detail [e.g. 27]. Here, each patient watched a short video clip, where a simple motor task is demonstrated. He/ She was then asked to repeat the demonstrated movement shortly after, and also rate his/her own performance on a dichotomous scale (yes/ no) in regard to observable deficits. A total of 15 motor symptoms could be evaluated within five repeated tasks:

1. Sitting on a chair: resting tremor (left and right hand), dyskinesia
2. Right hand pronation-supination: speed and amplitude of movement, dyskinesia
3. Left hand pronation-supination: speed and amplitude of movement, dyskinesia
4. Arising from a chair: resting tremor (left and right hand), dyskinesia
5. Walking down an aisle: resting tremor (left and right hand), dyskinesia

As the whole procedure was videotaped, the patient’s performance could later be evaluated by two independent raters. Whenever a discrepancy between the rater’s and the patient’s evaluation occurs (especially when the rater notices an impairment which the patient did not), ISAm was present. In a second step, for each motor task the severity of motor impairment is defined in accordance to the UPDRS-III scale (values range 0–4; 0 = normal/absent; 1 = mild; 2 = moderate; 3 = severe; 4 = unable to perform). Lastly, two global scores could be summarized: a total ISAm score (ISAm-total), which only includes all tasks with a discrepancy, and a total motor severity score. Both scores vary between 0–60 with higher scores indicating stronger impairment of self-awareness. Additionally, the total ISAm score was divided into subscores for ISA for hypokinetic symptoms (ISAm-Hypo for tremor and bradykinesia) and hyperkinetic symptoms (ISAm-LID) for further analysis.

The Montreal Cognitive Assessment [MoCA [33]] was used as a screening tool for global level of cognition. Each Patient’s cognitive performance was also intensively evaluated using a self-compiled neuropsychological test battery consisting of 13 tests. Following the guidelines for an MCI-Level II-Assessment by Litvan et al. (2012), at least two different neuropsychological tests were used for each of the five cognitive domains (see Table 1) [34].

**Table 1:** Overview of the applied neuropsychological tests.

Cognitive domain	Tests applied	Specific subtest
Attention/ Working memory	TAP	Sustained attention
	WMS-R	Digit span forwards/ backwards
Executive Functioning	RWT	Lexical verbal fluency
		Alternating lexical verbal fluency
		Semantic verbal fluency
		Alternating semantic verbal fluency
	Trail Making Test	Parts A+B
Language	WAIS-IV	Vocabulary
		Similarities
Memory	VLMT	Recalled words after 30 minutes
	ECFT-MI	Recognition Trial
Visuospatial Ability	WMS-R	Spatial Span forwards/backwards
	ECFT-MI	Matching Trial

Abbreviations: ECFT-MI, Extended Complex Figure Test–Motor Independent Version; RWT, Regensburg verbal fluency test; TAP, test battery for attention; VLMT, Verbal learning and memory test; WAIS-IV, Wechsler Adult Intelligence Scale–Fourth Edition; WMS-R, Wechsler Memory Scale-Revised.

Depression scores were assessed by using the BDI-II. Dispositional mindfulness was assessed using the Five Facet Mindfulness Questionnaire–German version [FFMQ-D, score range 0–195 [35]] with higher scores indicating higher levels of mindfulness. Here, patients rated their responses on a 5-point Likert scale with each item ranging from 1 (“never or rarely ever true”) to 5 (“very often or always true”). The FFMQ-D total score was also divided into five additional subscores, each representing another facet of mindfulness: Observing, describing, acting with awareness, non-judging of inner experience, non-reactivity to inner experience. Observing concerns the attention on external or internal stimuli. Describing assesses someone’s ability to express their own perception, including experiences, thoughts, and emotions. Acting with awareness refers to the capability of paying attention to the present moment. Non-judging of inner experience describes the degree to which persons label and rate their thoughts, emotions, and feelings as either good or bad. Finally, non-reactivity to inner experience means the individual’s tendency to react to their own thoughts, feelings, and emotions. It has to be noted though, that previous psychometric studies reported a four-factor structure (instead of five factors) for samples of non-meditators or clinical samples [36, 37]. Since this study included only mediation-naïve patients, the facet observing will be included, but interpreted with caution.

### Statistical analysis of behavioral and clinical measures

Behavioral and clinical data was analyzed using SPSS, version 27.0 (IBM Corp., Armonk, New York). Level of significance was set at 5% for all outcomes.

For the total scores of ISAm and motor symptom severity, intraclass correlation coefficients [ICC; absolute agreement, average-measures] were calculated to determine the degree of agreement between independent raters. Neuropsychological raw scores were converted into z-scores according to respective normative data (except for ECFT-MI Matching trial, which does not allow to calculate a definitive z-score). A global mean cognition z-score [similar to 38] was calculated using the calculated z-scores of all tests’ total scores listed in Table 1 (except ECFT-MI Matching trial due to its normative data).

All ISAm scores were not normally distributed. Therefore, non-parametric statistical methods were used (Spearman correlations). Correlation analyses were performed between ISAm scores and demographic, neuropsychological and questionnaire data. As age and education likely influence the performance in neuropsychological tests, analysis of neuropsychological data was corrected for both variables to rule out confounding effects. After calculating Spearman correlations of the FFMQ-D with basic sample characteristics (age, education, LEDD, MoCA score, UPDRS motor score, and global cognition z score), years of education and UPDRS motor scores were selected as covariates because of their respective highly significant test results. Therefore, correlation analyses of ISAm scores and mindfulness scores were corrected for the influence of education and motor severity. Due to the mostly exploratory character, no correction for multiple testing was performed.

To statistically determine the level comparability of the current study and previously published work by Maier et al. (2016), the two one-sided t-tests approach for equivalence testing with the variables age, education, LEDD, UPDRS-III scores and depression scores were performed using Microsoft Excel. The equivalence bounds were set at a benchmark of *d* = .20 (in accordance to Cohen’s *d* [39]) to detect inequivalences of medium and large effect sizes [40].

### Imaging data acquisition

Structural and functional MRI data were acquired on a 3-Tesla Tim Trio MR scanner (Siemens Medical Systems) with a 32 channel headcoil at the Department of Psychiatry, University of Marburg. After the initial localizer scan, structural images were acquired using a high resolution T1-weighted magnetization-prepared rapid gradient-echo sequence. The following parameters were utilized for this scan: 1 mm slice thickness, 0 mm gap, TR/ TE: 1900 ms/ 2.52 ms, matrix size of 256×256 voxel, field of view (FOV) 256 mm. Subsequently, a resting-state functional MRI scan was performed with a whole brain echo planar imaging (EPI) sequence and the following parameters: 490 volumes were obtained using 3-mm slice thickness, 0 mm gap, TR/TE = 1040/30 ms, flip angle = 55°, 70×70 voxel matrix size, FOV 210 mm [41–43].

### Pre-processing and analysis of imaging data

Preprocessing and analysis of resting-state functional MRI data was carried out using the CONN toolbox [44] and SPM12 (https://www.fil.ion.ucl.ac.uk/spm/). Preprocessing was performed according to CONN’s default preprocessing pipeline. Following coregistration, T1-weighted structural images were segmented and normalized to a template in Montreal Neurological Institute (MNI) space with a 1 x 1 x 1 mm resolution. T2*-weighted functional images were spatially realigned after coregistration and normalized to a MNI template with a 2 x 2 x 2 mm resolution using direct spatial segmentation and normalization. Functional scans were spatially smoothed using a 6 mm full-width half-maximum (FWHM) Gaussian filter. Outliers were defined using the artifact detection tool implemented in CONN. Here, outliers are identified using the observed BOLD signal and subject-motion in the scanner. Outliers were defined as a framewise displacement of at least 0.9 mm or a change of the global BOLD signal above five standard deviations. The framewise displacement is calculated based on 140 x 180x 115mm bounding box around the brain and estimating the largest displacement among six control points. The global BOLD signal change is defined as a change in the average BOLD signal within SPM’s global-mean mask scaled to standard deviation units. Framewise displacement as well as the global BOLD signal are computed at each timepoint. To deal with outliers, a variable number of noise components (one for each of the identified outlier) is used as potential confounding effect to account for any influence of the previously identified outlier scans on the BOLD signal [45]. Further denoising steps were applied to functional scans, including high-pass temporal filtering and linear detrending, removal of physiological confounds using the aCompCor method [46]. We also performed mainly two quality assurance checks to judge reliability of the imaging data. First, we checked, if the normalization process of structural and functional data was successful by generating figures of the normalized and realigned images (for each subject separately, but also averaged across all subjects). Secondly, we used quality assurance reports generated in CONN to examine the following parameters: the number of valid and invalid scans, the maximum, and mean extent of motion, but also the maximum and mean change of the global BOLD signal for every subject and the total sample.

Seed-based whole-brain correlations (Fisher-transformed bivariate Pearson correlation coefficient) were calculated in CONN using seed regions of interest (ROIs) from the Harvard-Oxford atlas. Based on the results of Maier et al. (2016), the following seeds were included in the analysis: Inferior frontal gyrus left and right, medial frontal gyrus left and right, superior frontal gyrus left and right, precentral gyrus left and right, insular cortex left and right, anterior and posterior cingulate cortex [7]. Significant associations between each ISAm score (total, hypokinesia and levodopa-induced dyskinesia) and functional connectivity between a respective seed ROI and every other voxel were evaluated using a general linear model in CONN. Age, gender, LEDD, and UPDRS-III scores were included as covariates in each analysis. All results of these multiple regression analyses were corrected for multiple comparisons using family-wise error (FWE) correction at cluster level.

## Results

Sample characteristics of the 41 patients (22 female and 19 male) included in the present study are depicted in Table 2. In total, six of these 41 patients showed moderate symptoms of depression (classified as BDI-2 score between 20 and 28 points [31]). Initially, a subsample of 17 patients (six female and nine male) participated in a resting-state fMRI examination. Due to insufficient quality of the unprocessed images, data from one subject was excluded. Data from another patient was excluded due to severe depression, resulting in 15 patients (six female and nine male) to be included in the fMRI analysis. Because of its low sample size, this must be interpreted as a preliminary analysis. None of the patients included in the subsample showed moderate signs of depression. Sample characteristics of the subsample of 15 patients, who participated in a resting-state fMRI examination, are summarized in Table 3.

**Table 2:** Sample characteristics of all PD patients.

	N = 41	
Sample characteristics	Mean (SD)	Median (range)
Age in years	68.22 (8.44)	69 (46–83)
Hoehn & Yahr stage	2.24 (0.54)	2 (1–4)
UPDRS motor score	36.38 (13.37)	32 (7–71)
MoCA total score	27.00 (2.28)	27 (20–30)
LEDD in mg	737.76 (383.64)	605 (150–1747.50)
Education in years	14.17 (3.25)	13 (10–21)
Global cognition z-score	-0.15 (0.48)	-0.07 (-1.15–0.62)
BDI-II total score	11.83 (7.31)	11 (1–28)
FFMQ-D total score	137.46 (19.04)	141 (96–179)
FFMQ-D subscale Observing	26.27 (5.79)	26 (16–39)
FFMQ-D subscale Describing	27.71 (6.56)	27 (17–40)
FFMQ-D subscale Acting with awareness	29.20 (4.52)	30 (19–38)
FFMQ-D subscale Non-judging	31.44 (5.36)	32 (20–40)
FFMQ-D subscale Non-reactivity	22.85 (4.80)	23 (12–35)
TAP omissions	7.80 (7.47)	5 (0–28)
Digit span forwards	7.46 (1.43)	8 (4–11)
Digit span backwards	6.12 (1.62)	6 (3–10)
Spatial span forwards	7.07 (1.40)	7 (4–10)
Spatial span backwards	6.39 (1.28)	6 (4–9)
RWT lexical verbal fluency	16.93 (7.24)	16 (4–36)
RWT alternating lexical verbal fluency	19.95 (6.32)	19 (9–36)
RWT semantic verbal fluency	32.51 (10.43)	31 (7–54)
RWT alternating semantic verbal fluency	19.95 (5.51)	19 (11–31)
Trail Making Test B/A	2.43 (0.65)	2.28 (1.44–4.46)
WAIS-IV Vocabulary total score	42.37 (8.51)	43 (14–57)
WAIS-IV Similarities total score	26.05 (4.80)	27 (15–34)
ECFT-MI recognition score	14.32 (5.24)	13 (4–25)
ECFT-MI matching score	9.68 (0.61)	10 (8–10)
VLMT Verbal Learning Memory	7.56 (3.38)	7 (0–15)
(Number of recalled words after 30 minutes)		
**ISAm-PD test symptoms**		
Total symptom severity	9.20 (6.29)	8 (1–28)
Symptom severity hypokinesia	6.80 (5.46)	6 (0–28)
Symptom severity Dyskinesia	2.39 (3.60)	1 (0–15)
**ISAm-PD test awareness**		
Total ISAm severity	5.71 (4.69)	5 (0–19)
ISAm severity hypokinesia	4.05 (4.29)	3 (0–19)
ISAm severity dyskinesia	1.66 (2.95)	0 (0–15)

Abbreviations: BDI-II, Beck Depression Inventory-2; FFMQ-D, Five Facet Mindfulness Questionnaire–German version; ECFT-MI, Extended Complex Figure Test–Motor Independent Version; ISAm, Impaired self-awareness of motor symptoms; LEDD, levodopa equivalent daily dose; MoCA, Montreal Cognitive Assessment; RWT, Regensburg verbal fluency test; TAP, test battery for attention; UPDRS, Unified Parkinson’s Disease Rating Scale; VLMT, Verbal learning and memory test; WAIS-IV, Wechsler Adult Intelligence Scale–Fourth Edition; WMS-R, Wechsler Memory Scale-Revised.

**Table 3:** Sample characteristics of fMRI subsample.

	n = 15	
Sample characteristics	Mean (SD)	Median (range)
Age in years	64.33 (8.38)	66 (46–79)
Hoehn & Yahr stage	2.00 (0.38)	2 (1–3)
UPDRS motor score	33.43 (10.93)	34 (7–48)
MoCA total score	26.67 (2.26)	26 (24–30)
LEDD in mg	713.00 (383.75)	562 (150–1505)
Education in years	15.33 (3.42)	15 (10–21)
Global cognition z-score	-0.01 (0.44)	0.06 (-0.93–0.62)
BDI-II total score	9.53 (5.38)	10 (1–20)
FFMQ-D total score	141.00 (20.52)	146 (104–179)
FFMQ-D subscale Observing	27.47 (5.74)	28 (17–39)
FFMQ-D subscale Describing	27.00 (6.78)	26 (17–40)
FFMQ-D subscale Acting with awareness	30.27 (4.65)	31 (22–38)
FFMQ-D subscale Non-judging	31.67 (5.91)	32 (21–40)
FFMQ-D subscale Non-reactivity	24.60 (5.19)	25 (13–35)
TAP omissions	5.27 (5.99)	3 (0–22)
Digit span forwards	7.80 (1.42)	8 (6–11)
Digit span backwards	6.87 (1.81)	7 (4–10)
Spatial span forwards	7.20 (1.61)	7 (4–10)
Spatial span backwards	6.80 (1.42)	7 (4–9)
RWT lexical verbal fluency	18.13 (6.00)	18 (7–27)
RWT alternating lexical verbal fluency	20.60 (6.73)	20 (9–36)
RWT semantic verbal fluency	33.40 (10.29)	31 (19–50)
RWT alternating semantic verbal fluency	21.20 (5.89)	22 (12–29)
Trail Making Test B/A	2.49 (0.76)	2.25 (1.46–4.46)
WAIS-IV Vocabulary total score	43.93 (7.92)	44 (27–57)
WAIS-IV Similarities total score	27.87 (3.70)	28 (22–34)
ECFT-MI recognition score	15.20 (6.44)	15 (4–25)
ECFT-MI matching score	9.67 (0.72)	10 (8–10)
Verbal Learning Memory	6.93 (3.60)	7 (0–14)
(Number of recalled words after 30 minutes)		
**ISAm-PD test symptoms**		
Total symptom severity	7.47 (4.07)	9 (1–13)
Symptom severity hypokinesia	5.67 (3.62)	5 (1–11)
Symptom severity Dyskinesia	1.80 (2.27)	1 (0–7)
**ISAm-PD test awareness**		
Total ISAm severity	4.40 (4.00)	4 (0–11)
ISAm severity hypokinesia	3.00 (3.51)	2 (0–10)
ISAm severity dyskinesia	1.40 (2.00)	1 (0–7)

Abbreviations: BDI-II, Beck Depression Inventory-2; FFMQ-D, Five Facet Mindfulness Questionnaire–German version; ECFT-MI, Extended Complex Figure Test–Motor Independent Version; ISAm, Impaired self-awareness of motor symptoms; LEDD, levodopa equivalent daily dose; MoCA, Montreal Cognitive Assessment; RWT, Regensburg verbal fluency test; TAP, test battery for attention; UPDRS, Unified Parkinson’s Disease Rating Scale; VLMT, Verbal learning and memory test; WAIS-IV, Wechsler Adult Intelligence Scale–Fourth Edition; WMS-R, Wechsler Memory Scale-Revised.

Both the total sample as well as the subsample appear to be comparable due to their similar demographics and characteristics. For a summary of statistical test results to check the comparability of the current study sample and the sample analyzed by Maier et al. (2016) see S1 Table. To summarize these results, both samples only demonstrate equivalence in regard of age, but not in regard of education, LEDD, UPDRS-III or depression scores.

### Impaired self-awareness of motor symptoms and its clinical correlates

Intraclass correlation coefficients were high for both ISAm total score (ICC = 0.892) and total motor severity score (ICC = 0.873). Thirty-six of 41 patients did not perceive at least one symptom. Therefore, ISAm was present in about 88% of patients. Detailed results for ISAm total score and all subscales can be found in Table 4. On the other hand, in 50 cases the patients noticed an impairment the independent raters did not acknowledge (in contrast to 130 unperceived motor symptoms). An overview of the ISAm-PD test results of the subsample is available in the supplemental data (S2 Table).

**Table 4:** ISAm-PD test results.

	Patients showing symptom	Number of motor symptoms	∑ Severity of motor symptoms	Patients not perceiving a symptom	Unperceived motor symptoms	∑ Severity ISAm
	N	N	N	N (%)	N (%)	N (%)
Dyskinesia	21	59	98	18 (85.71)	42 (71.19)	68 (69.39)
Tremor right hand	10	21	39	7 (70.00)	10 (47.62)	13 (33.33)
Tremor left hand	5	10	16	4 (80.00)	7 (70.00)	12 (75.00)
Bradykinesia	40	131	224	27 (67.50)	71 (54.20)	141 (62.95)
Hypokinesia	40	162	279	31 (77.50)	88 (54.32)	166 (59.50)
Total ISAm	41	221	374	36 (87.80)	130 (58.82)	234 (62.57)

For ISAm-total scores, significant correlations were found with age (r = .415, p = .007), education years (r = -.410, p = .008), UPDRS-III values (r = .425, p = .006) and global cognition z-score (r = -.312, p = .047). ISAm-total scores also strongly correlated with total ISAm symptom severity (r = .809, p = < .001) as well as ISAm-Hypo (r = .778, p = < .001) and ISAm-LID (r = .346, p = .027). Regarding neuropsychological raw scores, correlations of ISAm-total with RWT verbal fluency (r = -.349, p = .025), ECFT-MI recognition total score (r = -.429, p = .005) and digit span backwards score (r = -.436, p = .004) were significant. However, we did not find any significant correlations with neuropsychological test scores or global cognition z-score after correction for age and education. There were no significant correlations between ISAm-total and depression scores.

ISAm-Hypo did not correlate significantly with neither age nor education years. Positive correlations were found with UPDRS-III values (r = .468, p = .002) as well as with ISAm-Hypo symptom severity (r = .726, p = < .001). Several negative correlations with neuropsychological raw scores were significant, including ECFT-MI recognition total score (r = -.314, p = .045), lexical verbal fluency (r = -.417, p = .005), alternating semantic fluency (r = -.360, p = .018) and digit span backwards (r = -.441, p = .004). None of these correlations remained significant after correction for age and education. ISAm-Hypo did not correlate with global cognition z-score. Additionally, ISAm-Hypo significantly correlated with depression scores (r = .358, p = .021).

**Fig 1:**
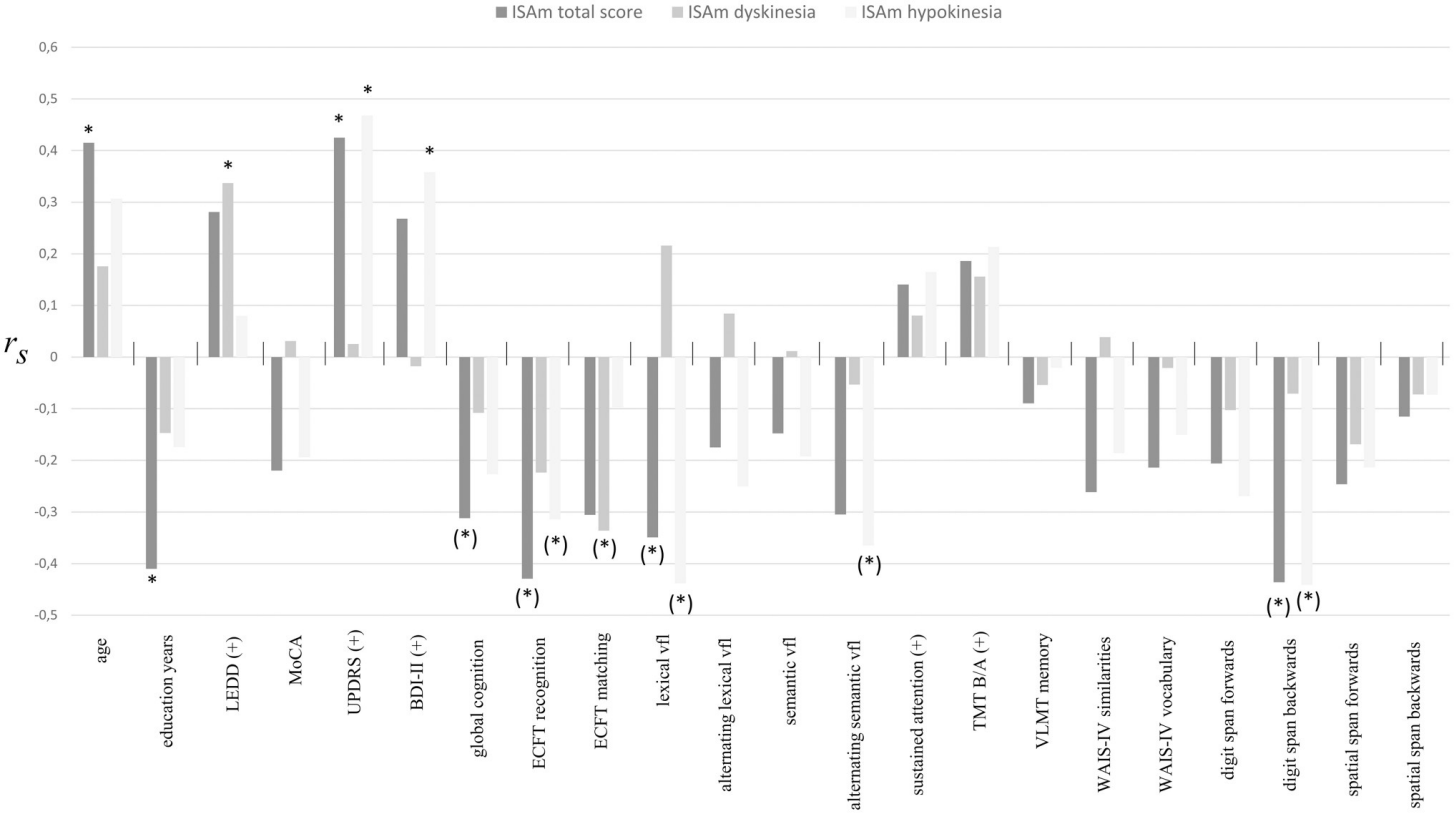
Spearman correlations between ISAm and demographic, disease-related and neuropsychological data. Abbreviations: BDI-II, Beck Depression Inventory; ECFT-MI, Extended Complex Figure Test–Motor Independent Version; LEDD, levodopa equivalent daily dose; MoCA, Montreal Cognitive Assessment; TMT, Trail Making Test; VLMT, Verbal learning and memory test; WAIS-IV, Wechsler Adult Intelligence Scale–Fourth Edition; vfl, verbal fluency; (+), higher scores indicate higher impairment or worse performance; *p < .05; (*) p ≥ .05 after correction for age and education years.

ISAm-LID did not correlate with age, education years, UPDRS-III values, depression, global cognition, or most neuropsychological test raw scores, except for ECFT-MI Matching (r = -.336, p = .032). After correction for age and education, there were no significant correlations between ISAm-LID and any neuropsychological raw score. The only significant correlations were found between ISAm-LID and ISAm-LID symptom severity (r = .860, p = < .001) as well as ISAm-LID and LEDD (r = .337, p = .031). It is worth mentioning, that ISAm-LID did not significantly correlate with ISAm-Hypo (r = -.175, p = .274). An overview of the ISAm correlation analyses results is illustrated in Fig 1.

### Imaging results

**Fig 2:**
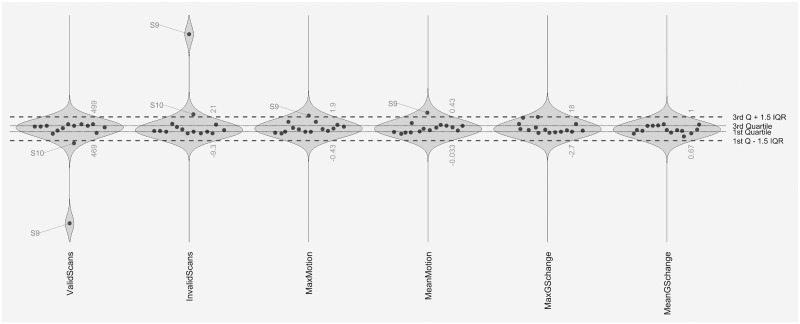
Overview of quality assurance measures for the resting-state fMRI subsample. Abbreviations: MaxGSchange, maximum change of the global BOLD signal; MeanGSchange, mean change of the global BOLD signal; MaxMotion, maximum amount of motion; MeanMotion, average amount of motion; Q, quartile; IQR, interquartile range, S, subject.

Quality assurance checks of the normalized and realigned structural and functional data were interpreted as sufficient for this preliminary analysis. The number of valid scans was M = 477.60 (SD = 31.52), while the number of invalid scans (or outliers) was M = 13.40 (SD = 31.52). On average, the observed motion was M = .22 mm (SD = .10). The largest motion was M = .81 mm (SD = .48). Changes of the BOLD signal was on average M = .83 (SD = .05). The largest change was M = 8.00 (SD = 4.41). Fig 2 gives an overview of these quality assurance variables for each subject included in the analysis. Only one subject clearly had a reduced number of valid scans, while also showing a slightly increased amount of motion. Another subject had a number of valid scans slightly below the mark of the 1.5 interquartile range, but did not show any signs of increased movement. As the potential influence of an increased amount of movement has been removed during the denoising process, no subject was excluded at this point to avoid an additional reduction of the sample size.

In total, multiple regression analyses between seed-based resting-state functional connectivity and ISAm scores revealed three significant clusters. Detailed results are depicted in Table 5.

**Table 5:** Voxel-wise regression results of ISAm scores and seed-based resting-state fMRI functional connectivity in PD patients (n = 15).

ISAm state	Seed region	L/R	Positive/negative	Cluster size	FWE corrected p-value	Peak (MNI)			Anatomical regions
						x	y	z	
ISAm-total	IFG	L	pos	104	.008	42	18	-4	Insular cortex right
									Frontal operculum right
									Frontal orbital cortex right
ISAm-LID	IFG	L	neg	77	.028	10	-6	48	Supplementary motor area
									ACC
									Precentral gyrus right
		R	neg	71	.041	12	-36	44	Precentral gyrus right
									PCC
									Precuneus
									Postcentral gyrus right

Abbreviations: LID, levodopa-induced dyskinesia; FWE, family-wise error; MNI, montreal neurological institute; IFG, inferior frontal gyrus; ACC, anterior cingulate cortex; PCC, posterior cingulate cortex.

**Fig 3:**
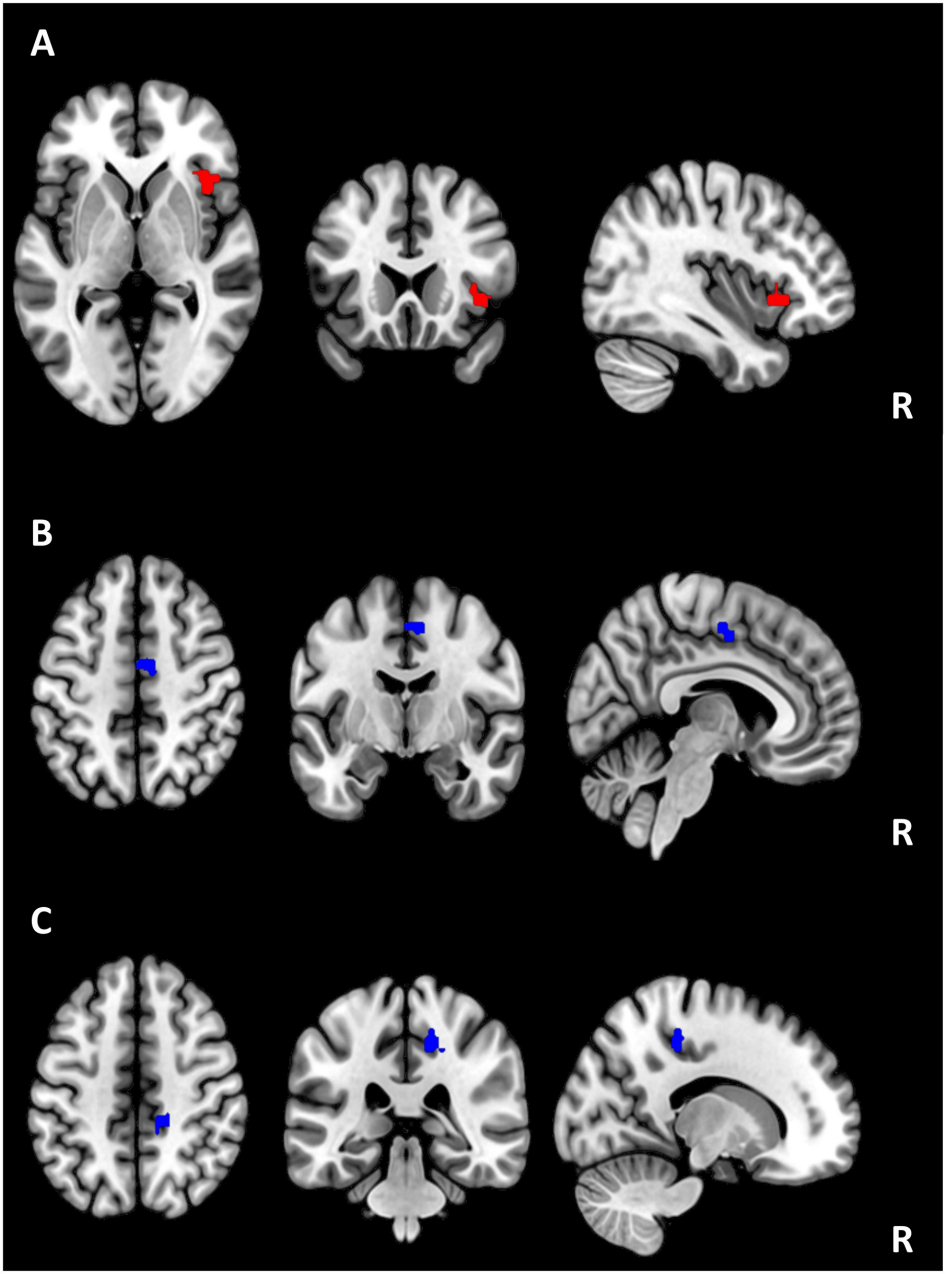
Associations between ISAm-total, ISAm-LID and resting-state fMRI functional connectivity. A) Positive association between ISAm-total scores and functional connectivity between the left IFG seed and the insular cortex. B) Negative association between ISAm-LID scores and functional connectivity between the left IFG seed and the supplementary motor area. C) Negative association between ISAm-LID scores and functional connectivity in between the right IFG seed and the precentral gyrus. All results were corrected for multiple comparisons using family-wise error (FWE) correction at cluster level. Abbreviations: ACC, anterior cingulate cortex; IFG, inferior frontal gyrus.

ISAm total scores were positively associated with the functional connectivity between the left IFG and the right IC, right frontal operculum, and right frontal orbital cortex. Significant negative associations were observed between ISAm-LID scores and the functional connectivity between both IFG seeds and the right precentral gyrus. These findings are illustrated in Fig 3. No significant associations with ISAm scores could be observed for the other seeds (after correction for family-wise error). There were no significant clusters regarding ISAm-Hypo.

### ISAm and mindfulness

To explore the concept of mindfulness in our study sample, we first correlated the FFMQ-D total score with basic sample characteristics. Hereby, we identified several significant correlations, namely with age (r = -.374, p = .016), education (r = .438, p = .004), the MoCA total score (r = .394, p = .011), the global cognition score (r = .385, p = .013), and the UPDRS motor score (r = -.481, p = .001). Only LEDD (r = -.063, p = .697) did not significantly correlate with the total mindfulness score.

**Fig 4:**
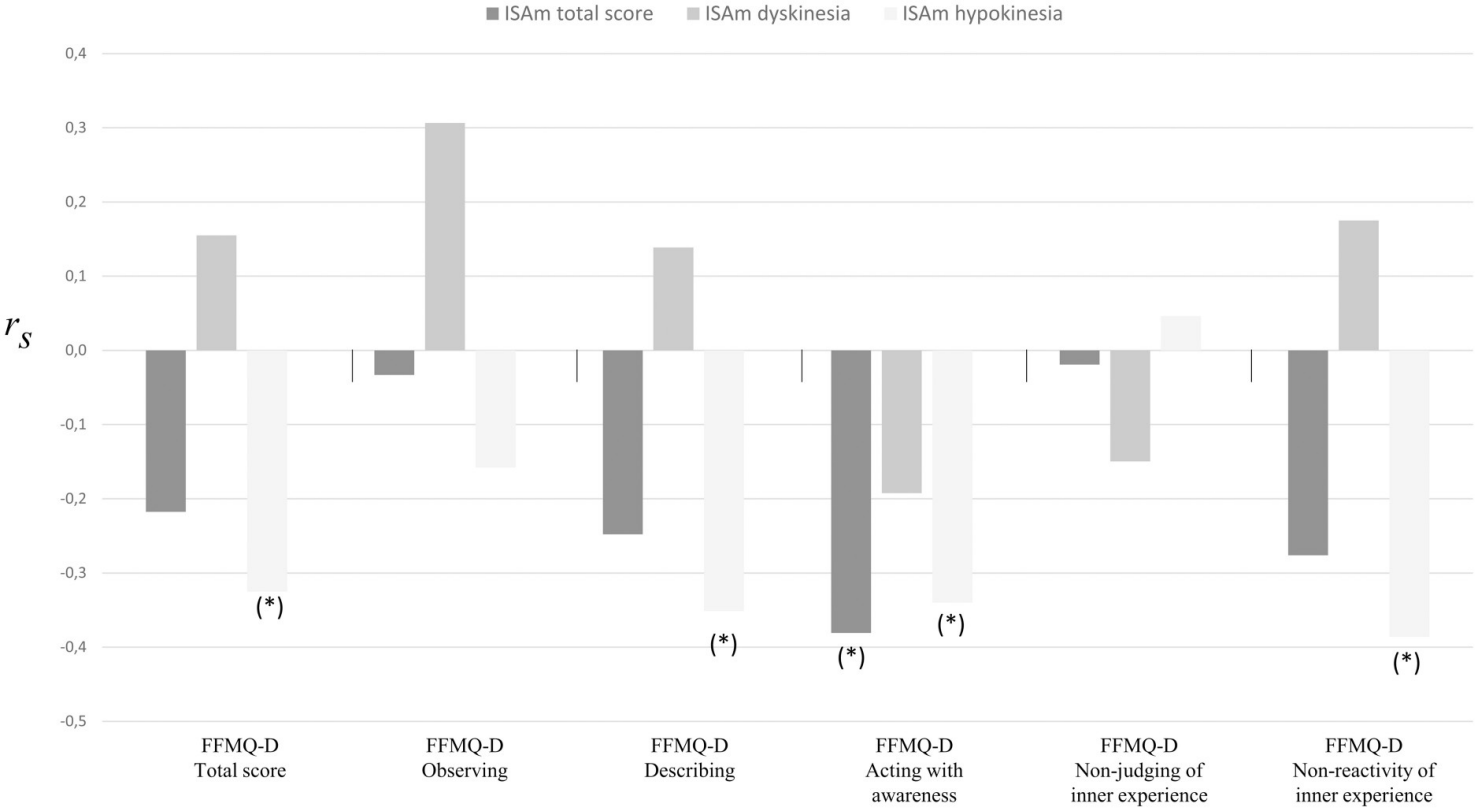
Spearman correlations between ISAm and FFMQ-D scores. Abbreviations: FFMQ-D, Five Facet Mindfulness Questionnaire–German version; *p < .05.

Initially, out of all six FFMQ-D scales (total score and five subscales), only the subscale acting with awareness was significantly correlated with ISAm-total scores (r = -.381, p = .014). ISAm-LID did not correlate with any subscale. Several negative correlations were found for ISAm-Hypo, including negative correlations with FFMQ-D total score (r = -.325, p = .038), subscales describing (r = -.351, p = .024), acting with awareness (r = -.381, p = .014) and non-reactivity to inner experience (r = -.386, p = .013). It is noteworthy, that subscale non-judging of inner experience and observing did not correlate with any of the three ISAm scores. However, after correcting for the influence of education and UPDRS motor scores, none of these results remained significant. Fig 4 summarizes the correlation analyses results of ISAm and mindfulness scores.

## Discussion

A few years ago, ISAm in PD including its clinical and neurobiological correlates based on FDG-PET have been evaluated by Maier et al. (2016) for the first time. In this study, we evaluated ISAm using the same structured approach [5] in another sample. For the first time, functional connectivity analyses were conducted to evaluate neural correlates of ISAm using resting-state fMRI. Additionally, the study broke new ground, being the first to explore a potential connection between ISAm and dispositional mindfulness.

### Clinical and neurobiological ISAm correlates

One goal of this study was to compare our data with the results of Maier et al. (2016), which presupposes a general comparability of the samples used. Unfortunately, despite a statistically proven equivalence in terms of age, both samples are rather disparate. Therefore, the general level of comparability is impaired. Also, there are a few differences, that need to be highlighted in the following: The total sample of Maier et al. (2016) consisted of 31 patients. Our total sample was larger with 41 patients included and showed only minimal differences concerning clinical and demographic variables, with slightly more advanced age and education, higher motor symptom severity, and LEDD. The current study included a higher proportion of female patients. Both samples were cognitively healthy according to mean values of their respective screening tools (Mini Mental State Examination or MoCA). While we performed all tests under patients’ regular medication, Maier et al. (2016) obtained neuropsychological and imaging data in the regular ON-state but measured motor symptom severity and ISAm in clearly defined medication ON- and OFF states.

#### ISAm-total and ISAm-Hypo

In total, a higher percentage of patients showed signs of ISAm (88%) in our sample compared to 61% of patients in the previous sample. Respectively, a similar case applied to ISAm-Hypo (78% vs. 41%). At least, a part of this increase might be due to an increase of age and motor severity in our sample, as both ISAm scores significantly correlated with their respective motor severity score. ISAm-total also correlated with the patients’ age. The latter has to be highlighted, as Maier et al. (2016) only found a positive correlation between age and ISAm-total in the OFF-state, but not in the ON-state. It has to be noted, that similar to Maier et al. (2016), we also did not correct for multiple testing. Therefore, the results must be interpreted within a highly explorative context.

Moreover, Maier et al. (2016) did not find any significant correlations of ISAm-total and any clinical or neuropsychological score. ISAm-Hypo significantly correlated with higher disease duration and worse verbal fluency (uncorrected). However, in our study both ISAm-total and ISAm-Hypo correlated with nearly the same neuropsychological raw scores (RWT lexical verbal fluency, ECFT-MI recognition, and digit span backwards). Initially, ISAm total score did correlate with global cognition z-score. In contrast, ISAm-Hypo score did not correlate with the global cognition z-score. However, similar to the study of Maier et al. (2016), none of the correlations with neuropsychological scores or global cognition remained significant after correction for age and education.

For ISAm-total, voxelwise regression analysis revealed a significant positive cluster for the left IFG seed, which included the right IC, right frontal operculum, and right frontal orbital cortex. This provides a link between ISAm and the prefrontal cortex and the insular cortex and surrounding structures, which have previously been linked with processes of reward learning, language, and empathy [47, 48]. In the general population, the orbitofrontal cortex has been assumed to be involved with decision making processes and reward learning [e.g. 49, 50]. In regard to self-awareness, our results are mostly in line with previous findings concerning neuroanatomic correlates (including the orbitofrontal cortex, the IFG and the IC) of ISA in Alzheimer’s disease [8]. Both the right IFG and the IC were also linked with anosognosia in stroke patients [51]. The right IFG is typically involved in motor inhibition processes [52, 53]. Therefore, it is surprising to identify functional correlates for the left IFG only. This raises the question of a potential functional reorganization, with a stronger connection between the left IFG and the right orbitofrontal cortex as well as the right insula and its surroundings. To the best of our knowledge, neither the orbitofrontal cortex, nor the right frontal operculum have been directly associated with impaired self-awareness of motor symptoms in PD. On a basic level, we assume, that the involvement of the left IFG might compensate for the missing involvement of the right IFG to maintain a connection between frontal regions and the insular cortex. Here, we identified a positive cluster indicating, that better connectivity correlates with a stronger impairment of self-awareness. Therefore, the left IFG (and its potentially reorganized connections) might be involved in maintaining or even increasing the degree of motor symptom severity misjudgment in patients with Parkinson’s Disease. Based on our results, no causality can be determined. Similarly, an increased involvement of the left IFG might also be an effect due to higher impairment of ISAm. More research might be necessary to clarify this hypothesis of functional reorganization. Also, due to the confounding nature of summarizing ISAm-Hypo and ISAm-LID to a global ISAm score, this must be interpreted with caution.

ISAm-Hypo, especially in earlier stages of PD, is expected to occur, because of symptoms being too mild and not causing enough impairment in daily life [6], which might be partly due to dopaminergic medication. To some extent, our results support this theory as ISAm-Hypo correlated with UPDRS motor severity score, and we also did not find any neural correlates for ISAm-Hypo in this sample of patients. Interestingly, ISAm-Hypo also significantly correlated with depression scores. For a long time, depression has been associated with altered self-awareness [54]. In the main sample, six patients showed signs of moderate depression. Our results suggest that ISAm-Hypo in our sample might be slightly confounded by depressive mood. However, even after including depression scores as a covariate, no significant results were identified.

#### ISAm-LID

In our sample, ISAm-LID was present in 85% of patients with dyskinesia, which is about the same proportion as 81% of patients in the sample of Maier et al. (2016) [7]. In our study, ISAm-LID did not correlate with any demographic or disease-related variables. There was no significant correlation between ISAm-LID and any neuropsychological score (after correction) in either of the two studies compared. It is especially noteworthy, that ISAm-LID did not correlate with global cognition or tests measuring executive functioning. Particularly, executive functioning has been hypothesized to be related to ISAm-LID [55]. However, as we did not apply a GO-NoGO-Task like Palermo et al. (2018), the comparability is limited. In both samples, ISAm-LID correlated with LEDD, and also highly correlated with ISAm-LID symptom severity. This might further support the claim, that dyskinesias are under high risk of being inadequately perceived.

On a neurobiological level, we found evidence for a role of functional connections between the left and right IFG and cingulate areas, the pre- and postcentral gyri, the supplementary motor area and the precuneus for ISAm-LID, which previously have been associated with the development of LID in PD [56–59] or functions of bodily awareness [59]. These findings connect the hypothesized main hub involved in the interpretation of dyskinetic movements [13] with areas being involved in the general development of LID. This further supports the hypothesis regarding mesocorticolimbic overstimulation caused by dopamine replacement therapy to play an important part in the pathogenesis of ISAm-LID [e.g. 12, 14]. Interestingly, our analyses were performed with several covariates like LEDD and age to reduce potential confounding effects indicating, that chronic overstimulation might not be the only factor to be considered here.

While the presented clinical data does not suggest, that ISAm-LID and executive dysfunctioning are mostly intertwined, the neurobiological results of this study further support existing theories surrounding the pathological mechanisms of ISAm-LID. We identified a negative association between the connectivity of bilateral inferior frontal gyri and the cingulate cortex and ISAm-LID, which are congruent with findings by Palermo et al. (2018) concerning a reduced activity of the anterior cingulate cortex to play an important part in the appropriate monitoring and integration of one’s movements. ISAm-LID has previously been linked to different brain regions including several frontal regions and the (anterior) cingulate cortex [7, 13] with the latter being highlighted as a potential main hub for the interpretation of dyskinetic movements [13, 14, 55]. Results of the present study support these findings using resting-state functional connectivity. Therefore, more severe signs of ISAm-LID might be present if connectivity between frontal regions and cingulate areas is reduced.

On a functional level, the aforementioned brain areas were identified to be part of a wider cognitive control network, also known as the “task positive” network [60, 61]. It comprises areas like the ACC and pre-supplementary motor area, the dorsolateral prefrontal cortex, the inferior frontal junction, the anterior IC as well as the dorsal pre-motor cortex and the posterior parietal cortex [e.g. 62]. This functional network is involved in controlling or coordinating multiple domains of cognitive control and executive functioning (e.g. attention, inhibition and planning) [63]. This indicates, that ISAm-LID in PD might partly result from reduced functional connectivity between areas forming the cognitive control network, which impairs the ability of self-monitoring and feedback integration.

### ISAm and mindfulness

First, we wanted to better understand the basic role of mindfulness in our sample. Interestingly, mindfulness correlated with education, as well as the MoCA total score and the global level cognition. This indicates a strong association with general level of cognitive functioning for PD patients. It is unclear, if this is different from the general population as mindfulness samples often tend to consist of highly educated people [64]. Total mindfulness also inversely correlated with age and motor symptom severity according to the UPDRS-III. Results of our sample might indicate that older and more impaired patients are less mindful. In a previous study, mindfulness was identified as a mediating factor between age and positive affect in the general population [65]. This is in line with the general positive effect of mindfulness on mental health [17]. Based on a survey of 5000 patients, van der Heide and colleagues also hypothesized a negative effect of stress on both motor and non-motor symptoms and suggest the conduction of further studies to establish the usefulness of mindfulness or other stress-alleviating interventions in PD [66].

To better understand the connection of ISAm and mindfulness, the diversity of the associations of ISAm scores and mindfulness facets might be of particular interest. At first, all mindfulness facets correlated with ISAm, except for FFMQ-D subscales non-judging of inner experience and observing. Initially, ISAm-total only correlated with FFMQ-D subscale acting with awareness, which refers to the person’s ability to be fully present in the current moment with regard to internal and external stimuli and therefore avoiding automatic pilot [67]. None of the mindfulness scales correlated with ISAm-LID. Also, ISAm-Hypo negatively correlated with FFMQ-D total score and subscales describing, acting with awareness and non-reactivity to inner experience. All results turned out to be non-significant after correcting for years of education and UPDRS scores. This implies, that in the current study the observed correlations of ISAm and mindfulness can be explained by correlations of mindfulness with basic sample characteristics. Based on our results, a connection of ISAm and dispositional mindfulness cannot be supported at this moment. However, we recommend to further examine possible associations in another sample of patients to get a better understanding regarding the presence or absence of a possible link.

### Limitations

We only assessed ISAm during the patients’ daily medication intake (ON-state). Moreover, we did not perform OFF-state measurements, as this would likely have been too burdensome for the patients as part of a larger clinical trial, from which this data was taken. Secondly, only a relatively small subsample (15 of 41 patients) was able to undergo a resting-state fMRI scan. Therefore, we have referred to it as a preliminary analysis with limited generalization of our results. Lastly, while we included an elaborate neuropsychological test battery, we did not include a GO-NoGO-task, which somewhat limits comparability to other studies in the field. To specifically study the possible connection of ISAm, executive functioning and the role of the ACC, future studies should therefore include a GO-NoGO-task. It has to be noted, that we compared data generated by two different imaging methods (fMRI vs. FDG-PET). Therefore, it can also be of further interest to combine MRI and FDG-PET imaging methods in another cohort of PD patients. On a general level, the comparability of clinical data with previous work conducted by Maier et al. (2016) is limited. As the clinical and neuropsychological results were not corrected for multiple testing (in both studies), all results must be interpreted within an exploratory context.

## Conclusions

In conclusion, the results of this study are in line with and sensibly complement the existing literature on the phenomenon of ISAm in PD. On a descriptive level, clinical correlates in our independent and slightly larger sample mostly matched the results from Maier et al. (2016) with few exceptions like age being correlated with ISAm-total in the ON-state. Seed-based functional connectivity analysis revealed functional correlates of ISAm and ISAm-LID, which mainly included an involvement of the IFG, insula, precentral cortex, the ACC and medial frontal gyrus, and therefore supported the hypothesis of cingulate areas being involved in the development of ISAm-LID by Palermo et al. (2018). Lastly, we explored the connection between ISAm and dispositional mindfulness. Our results do not suggest a possible connection between both constructs.

## Supporting information

S1 Table One-sided t-test results for equivalence testing of basic demographics and characteristics to assess comparability of the main study sample with the sample of Maier et al. (2016). Abbreviations: BDI-II, Beck Depression Inventory-2; LEDD, levodopa equivalent daily dose; UPDRS, Unified Parkinson’s Disease Rating Scale; *, significant finding.
(PDF)

S2 Table ISAm-PD test results of the fMRI subsample (n = 15). (PDF)
