# Supplementary material for: Exploring impaired self-awareness of motor symptoms in Parkinson’s disease: Resting-state fMRI correlates and the connection to mindfulness
Source: PLoS One. 2023 Feb 24;18(2):e0279722. doi: 10.1371/journal.pone.0279722 (PMC9955618; doi:10.1371/journal.pone.0279722)
Supplement: S1 Table — Abbreviations: BDI-II, Beck Depression Inventory-2; LEDD, levodopa equivalent daily dose; UPDRS, Unified Parkinson’s Disease Rating Scale; *, significant finding. (PDF) [file pone.0279722.s001.pdf]

Supplementary Material

S1 Table. One-sided t-test results for equivalence testing of basic demographics and characteristics to assess comparability of the main study sample with the sample of Maier et al. (2016).

	Main study sample (n = 41)	Sample of Maier et al. (2016) (n = 31)	Group difference	
	M (SD)	M (SD)	T	p
Age in years	68.22 (8.44)	65.13 (6.51)	9.33	< 0.001*
Education in years	14.17 (3.25)	12.84 (4.38)	-1.46	.075
UPDRS motor score	36.38 (13.37)	22.13 (9.08)	3.17	.999
LEDD in mg	737.76 (383.64)	633.13 (371.83)	-.36	.36
BDI-II total score	11.83 (7.31)	10.55 (4.78)	-.67	.25

Abbreviations: BDI-II, Beck Depression Inventory-2; LEDD, levodopa equivalent daily dose; UPDRS, Unified Parkinson's Disease Rating Scale; *, significant finding
