# Supplementary material for: Exploring impaired self-awareness of motor symptoms in Parkinson’s disease: Resting-state fMRI correlates and the connection to mindfulness
Source: PLoS One. 2023 Feb 24;18(2):e0279722. doi: 10.1371/journal.pone.0279722 (PMC9955618; doi:10.1371/journal.pone.0279722)
Supplement: S2 Table — (PDF) [file pone.0279722.s002.pdf]

Supplementary Material

S2 Table. ISAm-PD test results of the fMRI subsample (n = 15).

	**Patients showing symptom**	**Number of motor symptoms**	**∑ Severity of motor symptoms**	**Patients not perceiving a symptom**	**Unperceived motor symptoms**	**∑ Severity ISAm**
	**N**	**N**	**N**	**N (%)**	**N (%)**	**N (%)**
Dyskinesia	9	21	27	8 (88.89)	16 (76.19)	21 (77.78)
Tremor right hand	7	14	21	6 (85.71)	9 (64.29)	12 (57.14)
Tremor left hand	2	3	3	1 (50.00)	1 (33.33)	1 (33.33)
Bradykinesia	15	43	61	6 (40.00)	18 (41.86)	32 (52.46)
Hypokinesia	15	62	85	9 (60.00)	28 (45.16)	45 (52.94)
Total ISAm	15	83	112	11 (73.33)	44 (53.01)	66 (58.93)
